# The Effect of Aging in Inhibitory Control of Major Depressive Disorder Revealed by Event-Related Potentials

**DOI:** 10.3389/fnhum.2016.00116

**Published:** 2016-03-30

**Authors:** Bing-Wei Zhang, Jing Xu, Yi Chang

**Affiliations:** ^1^Department of Neurology and Psychiatry, First Affiliated Hospital of Dalian Medical UniversityDalian, Liaoning, China; ^2^Translational Medicine Research Center of Nerve System DiseaseDalian, Liaoning, China

**Keywords:** depression, aging, inhibitory control, event-related potentials, N2, P3

## Abstract

Elderly depressed patients manifest pronounced executive dysfunction compared with younger subjects with depressive disorder. Aging-related brain changes may result in executive dysfunction in geriatric depression. We investigated the neural correlates of inhibitory control processing in depressed subjects at different ages using event-related potentials (ERPs). A equiprobable visual Go/Nogo task was used in 19 young (27.4 ± 5.0 years) and 18 elderly (70.8 ± 6.9 years) depressed subjects and their age-matched healthy controls (20 young subjects, 26.2 ± 3.7 years, and 18 elderly subjects, 68.1 ± 4.8 years). The responses were based on two types of equilateral triangular figures of upright (Go) and inverted triangle (Nogo). The elderly subjects exhibited later N2 and P3 latencies, and larger Go-N2 and P3 amplitudes, compared with the younger subjects. Further, the elderly controls displayed smaller P3 in the central and parietal regions, and yielded larger Nogo-P3 amplitude in the frontal region compared with younger controls. While the young depressed patients yielded smaller P3 amplitude than the controls across frontal, central and parietal regions, elderly depressed patients yielded smaller P3 than the elderly controls only in the frontal region. Our results suggest that the inhibitory control subprocesses are differentially affected by depression and aging. The stimulus response speed and the effort intensity of inhibition control are specifically impaired in the elderly depressed patients. And the diminished amplitudes of frontal P3 in the elderly depression imply a frontal dysfunction mechanism.

## Introduction

In addition to mood disorder, a broad range of cognitive deficits contributes to the susceptibility to and maintenance of major depressive disorder (MDD; Lee et al., 2012). Clinically, patients with MDD during advanced age show more pronounced executive dysfunction (Herrmann et al., 2007; Koenig et al., 2014), including a set of cognitive behaviors essential for normal mental processes such as decision-making, conflict resolution, error correction, and response inhibition (Alexopoulos, 2003). However, mild executive dysfunction occurs with normal aging (Keys and White, 2000; Brown et al., 2015), provoking questions about the concomitant effects of aging and depression on executive function. A neuropsychological study revealed that compared with depressed younger counterparts and healthy elderly subjects, depressed elderly patients had disproportionately poor scores in terms of inhibitory control and focused effort, while no age-depression interaction was found for tasks of selective or sustained attention (Lockwood et al., 2002). These studies imply that in addition to aging, other brain changes may be associated with development of executive dysfunction in geriatric depression. However, the neural basis of this disparity of executive dysfunction in adult depressed patient at different age group is unclear.

Inhibitory control, which involves initiation, active switching, and inhibition of overlearned responses, is a central component of executive function (Norman and Shallice, 1986). Neuroimaging studies have reported the involvement of prefrontal cortex (PFC), anterior cingulated cortex (ACC) and frontal limbic area in the inhibitory mechanism (Garavan et al., 1999; Luijten et al., 2014), which are reliably activated in subjects overcoming interference from incorrect but pre-potent response tendencies (Botvinick et al., 1999; Carter et al., 2000). Structural magnetic resonance imaging (MRI) and functional MRI studies revealed structural and functional abnormalities of frontal lobe in depressed patients (Drevets et al., 1997; Ballmaier et al., 2004; Andreescu et al., 2013; Ironside et al., 2015; Wise et al., 2016) and normal aging adults (He et al., 2013). Further, the MDD patients showed abnormal activation in frontal and anterior cingulated areas during inhibitory processing (Matthews et al., 2009).

Event-related potentials (ERPs) provide enhanced temporal resolution of brain activities whereas fMRI techniques have limited use in studies of cognitive processes unfolding in the sub-second range. Inhibitory control processes can be examined by ERPs with Go/Nogo tasks (Falkenstein et al., 1999). Two major ERP components have been consistently linked with the inhibitory processes. First, the Nogo-N2 is regarded as a phasic negative shift in Nogo compared with Go trials, with a maximum amplitude over frontocentral scalp locations around 200 ms post-stimulus (Eimer, 1993). Second, a positive deflection of around 300 to 600 ms poststimulus with a larger amplitude than Go-P3 or P3b in the frontocentral area, is known as Nogo-P3 (Bokura et al., 2001). The Nogo-N2 is located within the ACC area and mediates the cognitive top-down inhibition mechanism to suppress the incorrect tendency to respond at a processing stage prior to motor execution (Falkenstein et al., 1999; Bokura et al., 2001). Nevertheless, the Nogo-N2 in Go/Nogo task also reflected conflict monitoring rather than response inhibition (Nieuwenhuis et al., 2003; Randall and Smith, 2011). The Nogo-P3 is likely to be related to motor inhibition (Bruin et al., 2001; Burle et al., 2004). However, the Nogo-P3 is not related to the inhibition itself, but to the cancellation of the planned response (Randall and Smith, 2011). Although the precise cognitive processes of Nogo-N2 and Nogo-P3 components remain unclear, they are implicated in inhibitory control mechanism (Huster et al., 2013).

There are a few published ERPs reports describing inhibitory control processes in MDD patients using a Go/Nogo paradigm (Kaiser et al., 2003; Zhang et al., 2007; Ruchsow et al., 2008; Katz et al., 2010). In a study of middle-aged adult depression (mean age = 40.0 years), a reduction in early frontotemporal positivity in the N2 time window (polarity-inverted N2) has been reported in an auditory Go/Nogo task (Kaiser et al., 2003). In addition, depressed patients (mean age = 40.1 years) in partial remission showed a specifically reduced Nogo-P3 while the Nogo-N2 was unaffected when performing a hybrid flanker Go/Nogo paradigm (Ruchsow et al., 2008). In a previous study, we measured ERPs from the subjects with late-life depression (mean age = 68.7 years) and matched controls using a simple visual Go/Nogo task, and found a larger Nogo-N2 and smaller Nogo-P3 in the depressed group (Zhang et al., 2007). Katz et al. (2010), however, found a strongly reduced Nogo-N2 enhancement in depressed elderly participants (mean age = 73.4 years), with data pointing to more posterior areas of the middle frontal gyrus. The inconsistent findings across these studies might be due to age-related effects, medication effects, differences in adopted paradigms and severity of depression, warranting further investigation.

Given neuropsychological tests have revealed that interaction between aging and depression minimizes executive function and compromises inhibitory control in patients with MDD (Lockwood et al., 2002), we hypothesized that aging and depression would interactively modulate ERPs. To our knowledge, the effects of aging and depression on inhibitory functioning were not simultaneously investigated via neuroelectrophysiological studies. Accordingly, the aim of the present study was to analyze ERPs presentation of subjects with MDD in different age groups, with a particular focus on the inhibitory control processes via Go/Nogo task. To control the effects of aging and depression on the ERPs waves, young adults and elderly MDD patients were included along with their gender-, education-, and age-matched controls.

## Materials and Methods

### Participants

Twenty young adults (age range of 21–36 years) and 21 elderly adults (age range of 60–80 years) with MDD were recruited from the First Affiliated Hospital of Dalian Medical University. All the patients met Diagnostic and Statistical Manual of Mental Disorders, 4th edition (DSM-IV; American Psychiatric Association, 1994) criteria of MDD single episode (296.3×) based on a Structured Clinical Interview for DSM-IV by two experienced psychiatrists. Twenty young adults and 20 elderly adults with no current or past axis I psychiatric diagnosis were recruited by advertisement and served as control groups. The young controls and MDDs as well as the elderly controls and MDDs were matched for age, gender, and education. All participants were right-handed and naive to antidepressants before this study. The exclusion criteria for patient and control participants were: (1) head injury or neurological disorders; (2) history of substance abuse; (3) other medical illnesses compromising the central nervous system; (4) the Mini-Mental State Examination (MMSE; Cockrell and Folstein, 1988) scores below 24; and (5) hearing, vision, or motor impairment that precluded the behavioral task. Severity of depression was evaluated by a Chinese version of the 17-item Hamilton Rating Scale of Depression (HRSD-17; Hamilton, 1960), and severity of co-morbid anxiety was indicated by a Chinese version of the 14-item Hamilton Anxiety Rating Scale (HAM-A; Hamilton, 1959). One young patient, three elderly patients, and two elderly controls were excluded because of insufficient number of correct artifact-free trials. The final sample consisted of 19 young patients and 18 elderly patients, 20 young controls and 18 elderly controls (Table 1).

**Table 1:** **Clinical demographic and behavioral profile (Mean SD)**.

Group	Sex (m/f)	Age (years)	Education (years)	Course (months)	HRSD-17^b^	HAM-A^b^	MMSE	RTs^a,b^ (ms)	HR^a^ (%)	FAR^a^ (%)	*d*′^a,b^
YD	8/11	27.4 (5.0)	12.8 (3.2)	10.2 (12.2)	21.1 (2.1)	19.2 (2.8)	29.8 (0.4)	320.0 (58.4)	98.1 (2.2)	5.4 (4.3)	4.0 (0.6)
YC	9/11	26.2 (3.7)	15.0 (3.1)	—	1.8 (1.3)	0.9 (0.8)	30.0 (0.0)	299.2 (34.8)	98.7 (2.2)	4.4 (3.1)	4.2 (0.6)
ED	5/13	70.8 (6.9)	9.8 (4.3)	4.8 (3.9)	21.5 (3.0)	17.9 (2.8)	27.7 (2.0)	366.5 (70.2)	95.2 (5.7)	10.4 (8.5)	3.3 (0.7)
EC	7/11	68.1 (4.8)	10.1 (3.3)	—	1.5 (1.1)	1.5 (1.1)	28.4 (1.3)	338.2 (39.8)	98.1 (3.5)	6.9 (7.6)	4.0 (0.8)

*Note: YD, young depressions (n = 19); YC, young controls (n = 20); ED, elderly depression (n = 18); EC, elderly controls (n = 18); HRSD-17, 17-item Hamilton Rating Scale of Depression; HAM-A, Hamilton Anxiety Rating Scale; MMSE, the Mini-Mental State Examination; RTs, reaction times; HR, hit rate; FAR, false alarm rate; d′, sensory discriminability. ^a^Main effect of age was found (p < 0.05), ^b^Main effect of depression was found (p < 0.05)*.

The study protocol was approved by the Ethical Committee of Dalian Medical University (KY2013–36). All the subjects signed written informed consent after the experiment was fully explained.

### Stimuli and Task

An uncued visual Go/Nogo task (Zhang et al., 2007) was presented on a computer monitor by STIM-2 Software. The whole task consisted of 200 stimuli. Upright or inverted equilateral triangular figures were used as the stimuli, which were white on a black background, presented pseudo-randomly with equal probability on the screen (light degree = 60 cd/m2), and with a viewing distance of 150 cm. The stimulus size was approximately 2.8° horizontally and vertically. The duration of the stimulus was 50 ms and the inter-trial interval was set at 950 ms. Participants were instructed to press a button with the left or right thumb as quickly as possible for each upright triangle (Go), but inhibit their response upon seeing inverted triangles (Nogo). The thumb used was counterbalanced among the subjects (a half subjects used their left thumb and another half used their right thumb). The speed of response was stressed, as the subjects had to respond within 600 ms, and responses beyond this window were deemed incorrect. All the subjects underwent a short training session containing 20 stimuli to ensure that they understood the task correctly. The reaction times (RTs) for Go stimuli and response accuracy were measured.

### EEG Recording and ERP Analysis

Electroencephalography (EEG) was continuously recorded from Ag/AgCl electrodes in an elastic cap (10/20 system) with a left mastoid reference. The EEG recording sites were: FP1, FP2, F7, F3, Fz, F4, F8, FT7, FC3, FCz, FC4, FT8, T7, C3, Cz, C4, T8, TP7, CP3, CPz, CP4, TP8, P7, P3, Pz, P4, P8, O1, Oz, and O2. The electrooculogram (EOG) was recorded using two pairs of electrodes, one placed above and below the right eye, and another 10 mm from the lateral canthi. The electrode impedances were kept below 5 kΩ throughout the experiment. The EEG was amplified using a Neuroscan NuAmps system. Amplifier settings included a bandpass of 0.1~100 Hz and a sampling rate of 1000 Hz.

The SCAN 4.3 Software was used for off-line analysis. The EEG was re-referenced to the average of the left and right mastoids. The EOG artifacts were corrected using the method proposed by Semlitsch et al. (1986). The EEG was segmented into the epoch from 200 ms pre-stimulus to 800 ms post-stimulus and the baseline corrected to the mean amplitude 200 ms before the stimulus. The trials contaminated with artifacts greater than ± 100 μV were rejected before averaging. Single trials were visually inspected and those containing muscular or other artifact were excluded manually. We removed the trials with response times shorter than 100 ms, as they were assumed to reflect non-deliberate behavior. The artifact-free EEG epochs with correct response for Go and Nogo conditions were averaged respectively. Only the trials with correct response (button-press) for the Go condition and correct inhibition (no button-press) for the Nogo condition were averaged, and at least 50 trials were available for each subject and condition. The numbers of trials remaining for each group were shown in Table 2. For further analysis, the ERPs data were digitally low-pass filtered at 16 Hz using a zero phase-shift filter (squared Butterworth FIR filter, 24 dB/octave).

**Table 2:** **The number of trials remaining for averaging for each group (Mean (SD))**.

Trial-type	YD	YC	ED	EC	*F* value	*p*
Go-trials	88.4 (9.5)	79.4 (16.4)	82.6 (10.9)	79.0 (15.7)	1.97	0.13
Nogo-trials	85.3 (12.2)	77.8 (15.8)	81.1 (11.6)	77.4 (15.0)	1.32	0.28

*Note: YD, young depressions (n = 19); YC, young controls (n = 20); ED, elderly depression (n = 18); EC, elderly controls (n = 18)*.

Visual inspection of the grand average waveforms revealed typical N2 and P3 components in all the groups. Based on previous studies (Falkenstein et al., 1999; Zhang et al., 2007; Ruchsow et al., 2008), the grand average waves and topographies for each experimental condition, the peak amplitudes and latencies of N2 were measured at the Fz and Cz sites, and the peak amplitudes and latencies of P3 was measrue at the Fz, Cz and Pz sites. Stimulus-locked time windows of 150–250 ms were selected for N2 and 250–420 ms for P3. Local minima (N2) or maxima (P3) were used for peak picking, manual intervention was possible during the process to ensure that the computer did not make anomalous peak selections. To avoid N2 component potentially distorting P3 component, peak-to-peak analysis (subtracting the peak amplitude and latency of N2 from P3) were performed at frontal and central sites (Fz and Cz).

### Statistical Analysis

The behavioral data (i.e., RTs, hit rate (HR) and false alarm rate (FAR)) were analyzed using univariate analysis of variance (ANOVA). The between-subject variables included age (young, elderly) and depression (depressed, control). Errors caused by delayed responses (missing the RTs deadline) were not considered. Accuracy of target signal detection was assessed by calculating sensory discriminability (*d′*) from hit and false rates (Swets et al., 1978).

For the ERPs data, mixed design repeated measures ANOVA (rmANOVA) were conducted, including the between-subjects factors of age (young, elderly) and depression (depressed, control), and within-subject factors of trial-type (Go vs. Nogo) and electrode-site (Fz and Cz for N2, Fz, Cz and Pz for P3). Global analyses were followed by restricted between- and within-subject analyses for interpretation of significant interactions. The Greenhouse-Geisser corrections were adopted where appropriate (Jennings and Wood, 1976). *Post hoc* comparisons were conducted using the Bonferroni procedure. The correlation between the ERPs and clinical and behavioral data was assessed using the Spearman’s rank correlation coefficient.

## Results

### Clinical Data and Behavioral Performance

Table 1 presents clinical and behavioral data of all groups. There was no difference in age, gender proportion and educational levels between young depressed and control subjects, and between elderly depressed and control subjects (all *p* values > 0.1). Also, the episodes did not differ statistically between young and elderly depressed patients (*p* > 0.05). Significant differences in HRSD-17 and HAM-A (all *p* values < 0.05), but not in MMSE (*p* > 0.05) were observed between patients and controls.

The young subjects showed shorter RTs, higer HR, lower FAR and higher *d*′ value than the elderly subjects for significant age main effects (*F*_(1,71)_ = 12.45, *p* = 0.001; *F*_(1,71)_ = 4.22, *p* = 0.044; *F*_(1,71)_ = 9.29, *p* = 0.036; *F*_(1,71)_ = 6.90, *p* = 0.011, respectively). In addition, the ANOVA revealed significant depression main effects for RTs (*F*_(1,71)_ = 4.11, *p* = 0.046, control < depression) and *d*′ (*F*_(1,71)_ = 8.22, *p* = 0.005, control > depression), but not for HR and FAR (all *p* values > 0.05). There was no significant interaction between age and depression in any behavioral measures (all *p* values > 0.05).

### Event-Related Potentials

**Figure 1:**
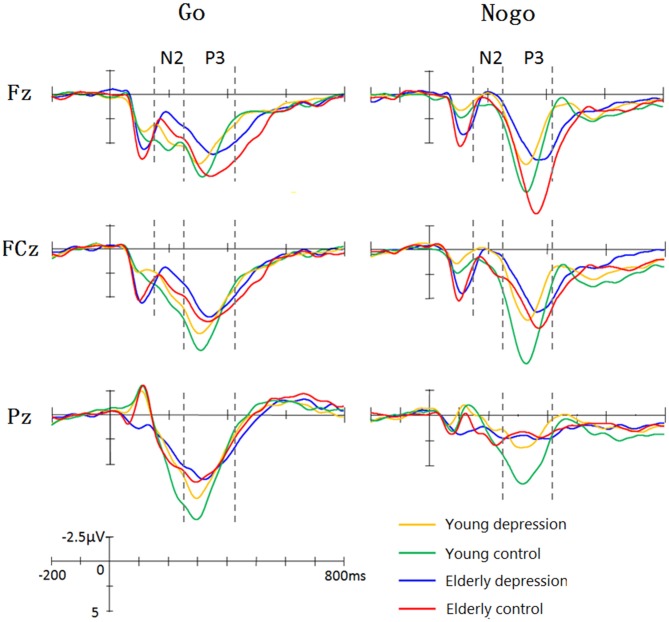
**Grand average event-related potentials (ERPs) elicited by Go and Nogo stimuli in four groups.** The young controls manifested larger P3 than young depressed patients across frontal (Fz), central (Cz) and parietal (Pz) sites. In elderly subjects, only Fz site showed differences between depressed and controls. The elderly subjects showed prolonged N2 and P3 latency and enhanced Go-N2 amplitude. The elderly controls elicited smaller P3 at central (Cz) and parietal (Pz) sites, whereas larger Nogo-P3 at Fz site compared with young controls.

**Figure 2:**
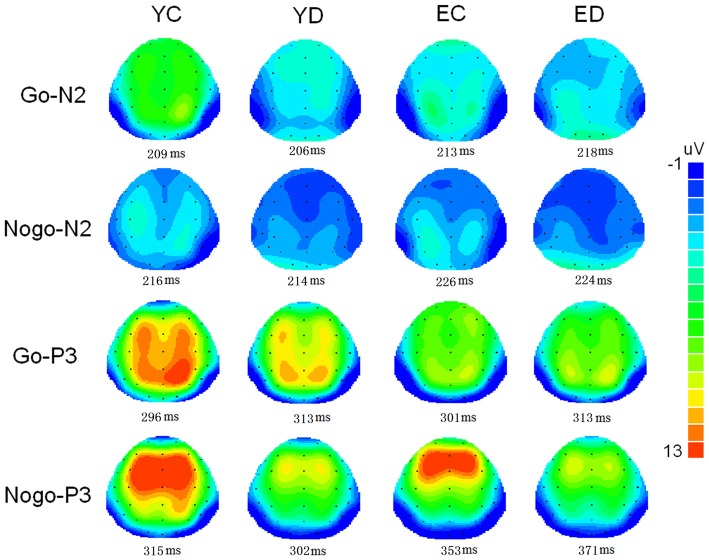
**Two-deminsional scalp topographic distributions of N2 and P3 elicited by Go and Nogo stimuli in four groups (YC, young control; YD, young depression; EC, elderly control; ED, elderly depression).** The peak latencies of N2 and P3 was selected across the four groups.

Figure 1 illustrate the grand-average ERPs over Fz, Cz and Pz sites in response to Go and Nogo trials. The topographic maps of the N2 and P3 time windows for each type trial are presented in Figure 2. For the sake of brevity, other ERPs components were not reported, and the effects not involving “age” or “depression” were not analyzed in detail.

#### N2 Amplitudes

As shown in Figure 1, Nogo stimuli evoked larger N2 component than Go stimuli in all the groups. Analysis of the N2 amplitude revealed a significant main effect for trial-type (−2.65 ± 7.63 μV for Nogo vs. 0.10 ± 7.64 μV for Go; *F*_(1,71)_ = 61.42, *p* < 0.001). As illustrated in Figure 1, young subjects showed smaller N2 compared with the elderly subjects in Go trials, whereas Nogo-N2 amplitude did not differ between young and elderly subjects. ANOVA yielded significant trial-type × age interaction effects (*F*_(1,71)_ = 5.33, *p* = 0.024). Depressed subjects demonstrated mildly larger Nogo-N2 amplitude than that of control groups, but the difference was not significant (*F*_(1,71)_ = 0.92, *p* = 0.341). No significant main effects and interactive effects were seen in between-subjects analysis in N2 amplitude (all *p* values > 0.1).

#### N2 Latencies

In regard to the N2 latency, there was a significant main effect of trial-type (*F*_(1,71)_ = 12.16, *p* = 0.001). As expected, Nogo stimuli yielded delayed N2 component than Go stimuli (217.16 ± 33.73 ms vs. 207.73 ± 32.48 ms). As shown in Figure 1, the elderly participants displayed later N2 peaks than the young, and ANOVA also revealed a main effect of age (221.20 ± 31.41 ms vs. 204.37 ± 31.41 ms;* F*_(1,71)_= 2.37, *p* = 0.012). The main effect of depression, trial-type × age and age × depression interaction were not significant (all *p* values > 0.5).

#### P3 Amplitudes

As illustrated in Figure 1, the Nogo-P3 was larger than Go-P3 in frontal and central regions, whereas Go-P3 was larger than Nogo-P3 in the parietal region of the four groups. The within-subject analysis yielded significant main effects of trial-type (9.84 ± 8.76 μV for Nogo vs. 11.01 ± 5.05 μV for Go;* F*_(1,71)_= 11.24, *p* = 0.001) and electrode-site (*F*_(1,71)_ = 31.47, *p* < 0.001) as well as a trial-type × electrode-site interaction which indicated the discrepancy of Go-P3 and Nogo-P3 distribution (*F*_(1,71)_ = 86.52, *p* < 0.001). *Post hoc* analysis demonstrated that Nogo-P3 amplitude was significantly larger than Go-P3 in Fz (11.74 ± 4.73 μV vs. 10.54 ± 4.54 μV) and Cz (11.11 ± 4.65 μV vs. 11.51 ± 4.88 μV) sites, whereas Go-P3 was significantly larger than Nogo-P3 in the Pz (10.98 ± 4.89 μV vs. 6.67 ± 4.21 μV) site (all *p* values < 0.05).

**Figure 3:**
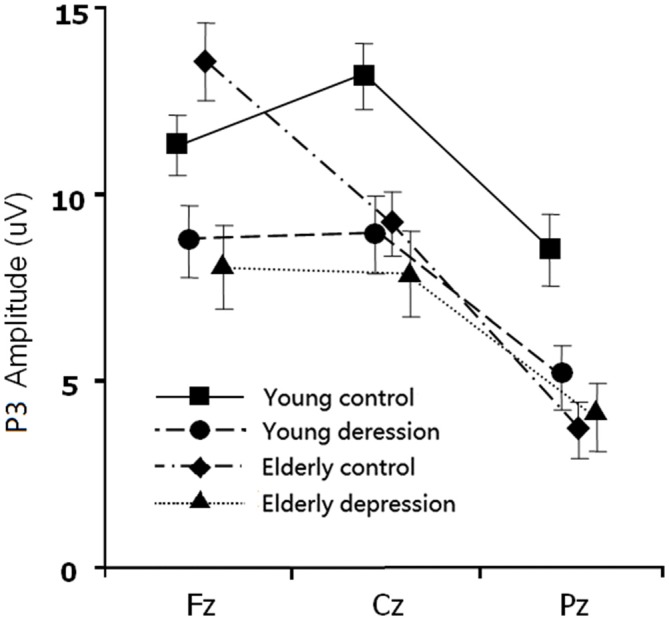
**The main effect and interaction effects of P3 amplitude.** Error bars represent standard errors.

The between-subject analysis revealed main effects of age (11.29 ± 4.84 μV for young subjects vs. 9.48 ± 4.83 μV for elderly subjects; *F*_(1,71)_ = 4.62, *p* = 0.035) and depression ((9.35 ± 4.96 μV for depression subjects vs. 11.46 ± 4.69 μV for controls; *F*_(1,71)_ = 6.33, *p* = 0.014), these main effects were qualified by age × electrode-site (*F*_(1.41,100.3)_ = 9.51, *p* = 0.001), depression × electrode-site (*F*_(1.41,100.3)_ = 3.50, *p* = 0.049), and age × depression × electrode-site interactions (*F*_(1.41,100.3)_ = 6.83, *p* = 0.005). To explain these interactions, further restricted ANOVAs were carried out. First, the ANOVA was performed on the young and elderly subjects separately to highlight the different pattern of P3 in depression and controls (Figures 1, 3). In the young subjects, a main effect of depression (12.46 ± 4.43 μV for young control vs. 10.07 ± 4.96 μV for young depressed; *F*_(1,37)_ = 4.32, *p* = 0.045) was observed (Figure 3), no other main effects or interactions reached significant level) all *p* values > 0.05). In the elderly subjects, however, there was no main effect of depression (*F*_(1,37)_ = 2.22, *p* = 0.146). A depression × electrode-site interaction was shown in elderly subjects (*F*_(1.42, 71)_ = 10.46, *p* < 0.001), and *post hoc* analysis indicated that the elderly depressed group yielded smaller P3 at frontal (Fz) site compared with the elderly controls (9.00 ± 4.77 μV vs. 13.24 ± 4.21 μV, *p* < 0.05; Figure 3). Second, the ANOVA was calculated in the control and depressed subjects separately to highlight the differences in the P3 pattern between the young and elderly subjects (Figures 1, 3). The P3 amplitude in young controls was significantly larger than in elderly controls (12.46 ± 4.43 μV vs. 10.37 ± 4.76 μV, *F*_(1,36)_ = 4.12, *p* = 0.049). Further, the main effect was qualified by age × electrode site (*F*_(1.33,50)_ = 16.06, *p* < 0.001) and age × trial-type × electrode-site interactions (*F*_(1.5,54.5)_ = 4.94, *p* = 0.019). *Post hoc* analysis revealed that the elderly controls displayed smaller P3 in the central (Cz; 9.64 ± 4.31 μV vs. 12.69 ± 3.72 μV) and parietal (Pz; 7.23 ± 4.36 μV vs. 11.55 ± 4.81 μV) sites (all *p* values < 0.05), but larger Nogo-P3 in the frontal (Fz) site (13.80 ± 4.42 μV vs. 11.79 ± 3.81 μV) compared with young controls (all *p* values < 0.05). In the depressed subjects, no age main effect was observed (all *p* values > 0.1).

#### P3 Latencies

As shown in Figure 1, elderly subjects yielded delayed P3 peaks compared with younger subjects. The between-subject analysis yielded significant age main effect (315.12 ± 37.35 ms for young vs. 350.42 ± 49 ms for elderly; *F*_(1,71)_ = 33.93, *p* < 0.001). The within-subject analysis revealed significant main effects of the trial-type (*F*_(1,71)_ = 57.35, *p* < 0.001) and electrode site (*F*_(1.38,97.9)_ = 24.09, *p* < 0.001). The Nogo-P3 showed later peak latencies than the Go-P3 (352.12 ± 40.75 ms vs. 328.31 ± 40.91 ms). Posterior P3 showed earlier peaks than the anterior sites (339.13 ± 44.35 ms for Fz site, 337.24 ± 46.08 ms for Cz site and 319.86 ± 47.46 ms for Pz). The trial-type effect (Go/Nogo effect) was further modulated by trial-type × age interaction (*F*_(1,71)_ = 11.07, *p* = 0.001). Significant age main effects were observed when ANOVAs were performed on the Go- and Nogo-P3 latency separately (young < elderly, all *p* values < 0.05). Remarkably, a depression main effect was found for the Nogo-P3 latency (340.49 ± 35.90 ms for controls vs. 355.20 ± 50.63 ms for depressed subjects; *F*_(1,71)_ = 5. 80, *p* = 0.027) but not for the Go-P3 latencies (*F*_(1,71)_ = 0.42, *p* > 0.5).

#### N2/P3 Peak-to-peak Amplitudes and Latencies

For the N2/P3 peak-to-peak amplitudes, the ANOVA showed significant main effects of depression (13.38 ± 5.49 μV for controls vs. 11.60 ± 4.13 μV for depressed subjects; *F*_(1,71)_ = 4.96, *p* = 0.029) and trial-type (14.08 ± 5.37 μV for Nogo vs. 10.93 ± 4.60 μV for Go, F_(1,71)_ = 43.37, *p* < 0.001). Similar to the analysis of the P3 amplitudes, the depression main effects of N2/P3 peak-to-peak amplitudes were further qualified by depression × electrode-site (F_(1,71)_ = 8.31 *p* = 0.004) and age × depression × electrode-site interactions (F_(1,71)_ = 9.12, *p* = 0.004). For the N2/P3 peak-to-peak latencies, the between-subject analysis yielded significant age main effect (115.87 ± 39.86 ms for young vs. 137.25 ± 48.09 ms for elderly; *F*_(1,71)_ = 7.80, *p* = 0.007). The within-subject analysis revealed significant main effects of the trial-type (136.12 ± 43.41 ms for Nogo vs. 116.65 ± 44.87 ms for Go, *F*_(1,71)_ = 13.97, *p* < 0.001). No significant age × depression interaction effect was shown on both N2/P3 peak-to-peak amplitudes and latencies (all *p* values > 0.1).

### Correlation Analysis

Correlation analysis between the clinical scale and ERPs measures (Nogo-N2 and Nogo-P3 in Fz site, Go-P3 in Pz site) in the young and the elderly depressed groups, respectively, demonstrated no significant correlation between HRSD-17 or HAM-A scores and ERPs measures (all *p* values > 0.05).

## Discussion

In the present study, the behavioral performance and ERPs components were assessed in young and elderly adults with MDD and their age-, gender-, and education-matched controls using a visual Go/Nogo task. The effects of depression and aging on the behavior and electrophysiology were analyzed. The younger subjects showed shorter RTs, higher HR, lower FAR and higher *d*′ indices than those of the elderly. The patients with MDD demonstrated longer RT and lower *d′* than in the controls. All the subjects displayed a distinct Go/Nogo effect, i.e., Nogo stimuli elicited larger frontal N2 and larger frontal P3 compared with Go stimuli. The Go-P3 was mainly distributed in the parietal region, whereas the Nogo-P3 showed “anteriorization” effects (Figure 2), replicating previous studies (Falkenstein et al., 1999; Bokura et al., 2001; Zhang et al., 2007; Randall and Smith, 2011). The young depressed patients yielded smaller P3 amplitude than young controls across frontal, central and parietal region (Figure 1). The elderly depressed patients yielded smaller P3 than the elderly controls only in the frontal region (Figures 1, 3). The elderly subjects showed delayed N2 and P3 latencies, and larger N2 (only for Go-N2) and P3 amplitude, compared with the young subjects (Figure 1). Further, the elderly controls elicited smaller P3 in the central and parietal regions, and yielded larger Nogo-P3 in the frontal region compared with young controls (Figures 1, 3).

### Effects of Age

The elderly subjects yielded longer N2 latency and larger Go-N2 compared to young participants, consistent with the decreased HR and *d*′ index in elderly subjects. Hence, the altered N2 latency in geriatric subjects may primarily be age-related, suggesting deficient pre-motor processes underlying response activation and inhibition in aged subjects, consistent with studies involving healthy elderly adults (Mudar et al., 2015) and depressed elderly subjects (Katz et al., 2010). Given the Go-N2 component may be associated with stimulus-response or response activation (Gajewski et al., 2008), the enhanced Go-N2 amplitudes in the elderly participants indicated that additional processing resources were required to activate the response signal in elderly subjects.

The elderly subjects displayed delayed P3 latency and N2/P3 peak-to-peak latency in both Go and Nogo trails compared with the controls, in line with a previous study (Vallesi, 2011). The prolonged P3 latency was also consistent with the defective behavioral data in the elderly groups, indicating that the elderly depressed participants were slower than the young participants in their responses to the stimuli.

The young controls displayed more robust P3 in the central and parietal regions but smaller frontal Nogo-P3 than those in the elderly controls. The parietal P3 (P3b) amplitude reflects the context-updating operations and subsequent memory storage and its latency reflecting stimulus evaluation time, which are essential for the cognitive processes (Polich, 2007). The frontal Nogo-P3 reflects the monitoring of successful outcome of the inhibition process (Schmajuk et al., 2006). Neurocognitive studies have reported altered patterns of brain activity associated with executive function in aged vs. younger adults (Turner and Spreng, 2012). Age-related activity during inhibitory control was observed in the right inferior frontal gyrus, and the elderly adults engaged the dorsolateral PFC as well as supplementary motor cortex and left inferior parietal lobule during working memory (Turner and Spreng, 2012). Therefore, the present behavioral and ERPs outcomes confirmed that altered cerebral cortex activity generally mediated normal aging during executive processing.

The elderly controls showed larger frontal Nogo-P3 than the young controls in this study, consistent with a previous study (Vallesi et al., 2009). However, no significant correlation of subject’s age with the Nogo-P3 amplitude was observed (Fallgatter et al., 1999). Mudar et al. revealed significantly reduced Nogo-P3 amplitudes in the aged compared with younger adults (Mudar et al., 2015). This discrepancy suggests a nonlinear relationship between inhibitory strength and Nogo-P3 amplitude considering that the tasks differed across these studies. Therefore, further investigations into mechanisms modulating the inhibitory strength are needed to test this hypothesis.

### Effects of Depression

The depressed subjects demonstrated mildly enhanced Nogo-N2 but did not reach significant level, supporting a previous study showing an unaffected Nogo-N2 (Ruchsow et al., 2008), but inconsistent with the results showing more negative Nogo-N2 in depressed groups (Kaiser et al., 2003; Zhang et al., 2007). In Kaiser et al.’s (2003) study, all the patients were middle-aged adults (mean age = 40.0 years) and treated with antidepressant medication at the time of the experiment, and the task they used was a modification of the auditory oddball paradigm. Our previous research (Zhang et al., 2007) adopted a same paradigm to the present study in elderly subjects, but eleven patients were taking antidepressant. Several studies demonstrated that medication *per se* has an impact on ERP amplitudes (e. g. Johannes et al., 2001). Therefore the divergence might be attributed to the differences in processing demands and the baseline demographic and medical profile across these studies.

The between-subject analysis revealed main effects of depression in P3 amplitude as well as N2/P3 peak-peak amplitude (depression < control). Additionally, our results revealed a depression main effect for the the Nogo-P3 latency (depression > control). While Nogo-P3 reflects response inhibition, Go-P3 reflects response activation, the activation of elements in an event categorization network that is controlled by the joint operation of attention and working memory (Kok, 2001). Therefore these results consistent with the clinical facts that depressed patients manifest both general attention impairment and executive dysfunction (Kindermann et al., 2000; Alexopoulos, 2003; Lee et al., 2012).

### Effects of Interactions

In the present study, the ERP measurements did not yield any first-order age × depression interaction, but showed significant age × depression × electrode-site interactions on P3 amplitudes and N2/P3 peek-to-peek amplitudes. The young depressed subjects manifested decreased P3 amplitude across frontal, central and parietal regions compared with the young controls, but in the aged subjects, depressed patients showed diminished P3 magnitude only in the frontal region, especially for the Nogo-P3. Diminished Nogo-P3 has been reported in young depressed (Ruchsow et al., 2008) and elderly depressed patients (Zhang et al., 2007). The Nogo-P3 originates within the ACC (Fallgatter et al., 2004; Gonzalez-Rosa et al., 2013), midcingulate cortex (MCC) and the pre-central region as well as the insulae (Huster et al., 2010). It is responsible for response monitoring and is connected to brain structures that regulate mood, emotion, thought and visceral responses (Schneider-Hassloff et al., 2015). Indices of ACC dysfunction are associated with adverse outcomes of geriatric depression (Alexopoulos et al., 2008). Importantly, the present study showed altered cerebral pattern of inhibitory between young and elderly depressed patients. The amplitude of P3 diminished more “anteriorly” in geriatric depressed patients than in young depressed patients, implying a mechanism of frontal dysfunction in geriatric depression.

Numerous studies suggested that executive dysfunction occurred in a considerable number of older individuals with MDD (Alexopoulos et al., 2000, 2008; Kindermann et al., 2000; Lockwood et al., 2002; Alexopoulos, 2003; Herrmann et al., 2007). Previous findings provide indirect support for the role of frontostriatal-limbic abnormalities in the executive dysfunction of geriatric depression (Kindermann et al., 2000; Hannestad et al., 2006; Zhang et al., 2007; Bobb et al., 2012). However, given the potential contribution of age on the brain regions related to executive function (He et al., 2013; Brown et al., 2015), and the dependence of different subcomponents of inhibitory control on different brain circuits (Huster et al., 2010, 2013), the present results suggest variable age- and depression-related deficits in the timing or rate of decline in function. Our results suggest that the response stimulus evaluation speed (indexed as Go-P3 latency) and the intensity of inhibition monitoring or evaluating process (indexed as frontal P3 amplitude) are impaired in the elderly depressed patients, whereas the pre-motor inhibition processes (indexed as N2 component) are modulated by aging but less likely by depressed status.

No significant correlation between ERPs measurements and the HRSD-17 was found, consistent with two previous studies (Kaiser et al., 2003; Ruchsow et al., 2008) but in contrast to another study (Zhang et al., 2007). These findings suggest that the inhibitory control deficit might be a disease-dependent feature in MDD patients.

This study is limited by the cross-sectional design involving distinct age groups. Therefore, further work is needed to investigate the role of age as a continuous variable across a larger number of participants. The feasibility of ERPs as biological markers of recovery in longitudinal studies needs to be evaluated in the future.

In conclusion, our findings suggest that the subprocesses of inhibitory control are differentially affected by aging and depression. The pre-motor inhibition processes are modulated by aging but less likely by depressed status. Specifically, the stimulus response speed and the effort intensity of inhibition control are impaired in the elderly depressed patients. And the diminished amplitudes of frontal P3 in geriatric depression imply a frontal dysfunction mechanism.

## Author Contributions

BWZ and JX carried out the studies, participated in collecting data, and drafted the manuscript. YC performed the statistical analysis and participated in its design. All authors read and approved the final manuscript.

## Conflict of Interest Statement

The authors declare that the research was conducted in the absence of any commercial or financial relationships that could be construed as a potential conflict of interest.
